# Feasibility and impact of a 1-minute daily functional exercise regimen prescribed to older adults by their primary care physician

**DOI:** 10.1016/j.pmedr.2020.101307

**Published:** 2021-01-04

**Authors:** Christopher N. Sciamanna, Matthew A. Ladwig, David E. Conroy, Kathryn H. Schmitz, Matthew L. Silvis, Noel H. Ballentine, Brandon J. Auer, Margaret K. Danilovich

**Affiliations:** a Penn State College of Medicine, Hershey, PA, United States; b Penn State University, State College, PA, United States; c Northwestern University, Evanston, IL, United States

**Keywords:** Primary care, Older adults, Physical function, Disability, Resistance training, Adherence

## Abstract

Despite the inverse relationship between physical activity (PA) and physical function, few older adults achieve PA recommendations. In response to observations that “lack of time” underlies reduced PA among older adults, recent work suggests even short bouts of PA can improve health and fitness. In addition, because they are frequently visited by older adults, an important conduit for PA promotion could be the primary care physician (PCP). However, most PCPs receive little training related to PA, rendering it difficult for them to offer meaningful counseling. Therefore, we explored the feasibility and impact of a PCP-prescribed one-minute daily functional exercise program, consisting of 30 s each of bodyweight push-ups and squats, among 24 patients 60 years of age or older. 42% of patients who were contacted started the exercise prescription and, over 24-weeks, completed approximately 114 sessions, while 75% completed at least half of the possible daily exercise sessions. As a group, the patients demonstrated increases in both maximal push-up and squat performance, though these increases plateaued following week-12. These preliminary results suggest that a PCP prescription of one-minute of daily functional exercise among older adult patients was feasible, acceptable, and effective for improving functional physical fitness. Given these findings, formal controlled research with recruitment from multiple clinics, random assignment to treatment conditions, and blinded assessments of objective functional physical performance should be pursued.

## Highlights

• 42% of older adult patients (≥60yrs) adopted a brief daily exercise regimen prescribed by their PCP.
• Over 24-weeks, the patients completed an average of 114 daily exercise sessions.
• Over 24-weeks, exercise performance increased by around 6.5 push-ups and 5.7 squats.

## 1 Introduction

For each decade after the age of 65, older adults lose the ability to perform, on average, an additional 2 chair-stands within 30 s (Rikli and Jones, 1999). This age-related loss of capacity to complete chair stands, a common activity of daily living (ADL), could increase the likelihood that individuals may need assistance to perform their daily activities. At the same time, these decrements in chair stand performance could be reduced by 50% simply by increasing PA (Paterson and Warburton, 2010, Tak et al., 2013). Despite these findings, older adults, or those who may benefit the most from PA, are often the least likely to accumulate enough of it (Kraschnewski et al., 2014, Tobi et al., 2012). Though the determinants of PA behavior are manifold, among the most frequent reasons older adults provide to explain their lack of PA is that they do not have enough time for it (Bethancourt et al., 2014, Centers for Disease Control and Prevention, 2008, Costello et al., 2011, Piercy and Troiano, 2018, Reichert et al., 2007). With this perceived barrier in mind, in this paper we report on an ultra-brief exercise program designed to circumvent time barriers while targeting functional strength and whether patients would adopt and adhere to such a program if directly prescribed by their primary care physician (PCP).

For years, PA guidelines have recommended at least 150 min of MPVA and two sessions of strength training weekly (Centers for Disease Control and Prevention, 2008, Piercy and Troiano, 2018). However, growing evidence suggests that far shorter periods of PA may also provide health and fitness benefits. For instance, Saint-Maurice and colleagues showed that MVPA bouts as short as 5-minutes were associated with decreased mortality (Saint-Maurice et al., 2018). Similarly, in a cohort of 416,175 adults, Wen and colleagues reported a 14% reduction in mortality for those doing 15 min of MVPA per day versus zero (Wen et al., 2011). This growing body of evidence is perhaps best reflected in the Physical Activity Guidelines for Americans (Centers for Disease Control and Prevention, 2008, Piercy and Troiano, 2018) where it is suggested that *any* PA is better than none, as the greatest benefits to health are observed among those who progress from doing “no“ PA to being “active, but not meeting guidelines” (i.e., a dose–response effect (Sadarangani et al., 2014)). These recommendations highlight the importance of providing not only safe and effective PA, but activities that people will adopt and adhere to.

Because older Americans make approximately 225 million visits to their PCP per year (National Center for Health Statistics, 2016), primary care could be an effective avenue to promote PA among older adults. However, despite this large number of visits, fewer than one-in-six include PA advice and counseling (Kraschnewski et al., 2013). Perhaps more concerning, the proportion of US PCPs providing PA counseling during visits decreased from 14.2% in 1995 to 11.3% in 2008, despite obesity rates nearly doubling over the same period. Lack of PCP time and training appear to be the most significant barriers to delivering PA advice and counseling (AuYoung et al., 2016). For instance, in their systematic review, Hebert and colleagues reported that having “too little time” to address PA was reported by PCPs in 14/19 studies and insufficient training in 8/19 (Hebert et al., 2012). These findings are less surprising considering that less than 15% of medical schools include PA in their curricula, and less than half of PCPs report any PA-related training (Garry et al., 2002, Cardinal et al., 2015). As a result, few PCPs report successfully promoting PA among their patients or endorsing the belief that their patients would become more active if provided with PA advice and counseling (Hebert et al., 2012, Walsh et al., 1999). Given the hurdles associated with disseminating PA into primary care, simple, effective, and time-efficient PA promotion approaches must be developed. Therefore, in this paper, we report the results of a quality of care improvement initiative that aimed to assess the impact of a PCP-prescribed brief functional exercise regimen on PA adoption, adherence, and physical performance among the older adult patients of the PCP.

## 2 Methods

### 2.1 Intervention development

#### 2.1.1 Frequency

We chose a daily exercise frequency to tie the exercise to the behavior of tooth brushing, which takes about the same amount of time as the prescribed exercises, and because research suggests that behaviors become habits more readily when repeatedly performed with similar timing and context (Beeken et al., 2017, Judah et al., 2018).

#### 2.1.2 Intensity

Because the intervention was intended to be delivered remotely, it was unlikely that we could tightly constrain exercise intensity, nor expect consistent vigorous-intensity exercise among an older adult sample. Additionally, we were concerned that vigorous-intensity exercise could present a health-risk for patients with unrecognized coronary artery or other diseases (American College of Sports Medicine, 2013). Therefore, we encouraged patients to work “hard” and complete “as many repetitions as possible”, but avoided the phrasing typically associated with high-intensity exercise programs, such as to provide “all out” or “110%” effort. These messages were reinforced daily during audio pre-recorded by the PCP.

#### 2.1.3 Time

Because we aimed to create a new habit for older adults, we focused on not exceeding the duration that most people brush their teeth (~60–90 s), arguably one of the most common health-related habits adults perform without reminders or external reinforcement (Delta Dental, 2014). Therefore, the daily exercises consisted of 30 s of push-ups and 30 s of squats with a 15 s rest period between the two exercises.

#### 2.1.4 Type

We prescribed only push-ups and squats for several reasons. First, push-ups and squats engage most large muscle groups (Hanson et al., 1995) and are involved in many activities of daily living, such as standing, walking, climbing, pushing, and pulling (Kraschnewski et al., 2014). Secondly, neither exercise required the purchase of equipment or substantial time for setup. Perhaps more importantly, reducing the number of exercises could ease dissemination by reducing complexity for both health care providers and patients (Berwick, 2003).

### 2.2 Participants

The current program was reviewed by the Institutional Review Board, who decided it was not consistent with the federal definition of *research* under 45 CFR 46.102(d) (Lynn et al., 2007). Instead, for several reasons, the project was deemed a quality improvement initiative, as opposed to a research study. First, the goal was to improve the health of patients with whom one of the authors (CNS) has an ongoing commitment to improve the local quality of care as the PCP of the patients in this program. Second, the intervention, while novel in its delivery, was not considered a new or unproven treatment, given the research base supporting the benefits of the exercises included in the intervention. Third, subjects were not randomized. Fourth, the project was not funded by an outside organization. Finally, the project has no fixed goal or end point - it remains ongoing with the first author (CNS) and his patients, it does not have a fixed methodology (i.e., adjustments to the program will be made if new evidence supports them), or population (i.e., patients can enter and leave the program at any time).

The individuals who participated were regular patients of the PCP and lead author (CNS) 60 years of age or older contacted by email to begin the exercise prescription. Although most patients had at least one medical diagnosis, the PCP contacted patients that maintained the functional physical capacity to exercise. The patients received up to 4 (with 72 h between) emails until they initiated the program or communicated their wish to opt-out of it. The exercise program was framed in the same manner as a typical pharmaceutical prescription, with a clear expectation that patients would complete the program daily and their results would be discussed during clinic visits (see Supplementary Materials for communications).

## 3 Procedures

### 3.1 Daily exercise message.

Emails to each patient from the PCP were sent at 6:00 AM daily and a reminder at 6:00 PM if no response was yet received. The day before receiving the first (i.e., baseline) survey to enter exercise performance data, each patient completed a short survey about current MVPA and exercise self-efficacy. The daily email included a link to a survey that included the following elements: 1) videos featuring the PCP demonstrating the exercises, modifications, and proper form, 2) a pre-recorded audio countdown timer (PCP-narrated; see Supplemental Materials for audio transcripts), and, 3) three-items for patients to enter their daily number of push-ups and squats completed in 30 s and whether they used modifications for push-ups. An example modification for push-ups was to perform them on the knees until 15 repetitions could be completed, followed by encouraging a transition to full push-ups. Alternatively, patients were shown a push-up modification using a staircase where they placed their hands on a stair at chest-level. Once able to complete 12 repetitions, they were encouraged to progress down one stair. A squat modification was provided where patients were encouraged to descend halfway (i.e., femurs at 45°), gradually increasing the distance descended until their femurs were parallel to the ground (i.e., 90° bend at the knees). Finally, for the first month, the surveys included measures of session perceived exertion and affective responses.

### 3.2 Baseline and last day of weeks-12 and-24 maximal 30 second exercise performance test

To provide more accurate estimates of maximal 30 s exercise performance, the instructions in the survey emailed on the first day of exercise and on the last day of weeks-12 (i.e., day 84) and-24 (i.e., day 168) emphasized maximal effort over each 30 s set of push-ups and squats. The same 3-items as the daily surveys were used to record maximal 30 s exercise performance.

### 3.3 Messages on first day following baseline, last day of weeks-12 and -24

To allow for recovery following each maximal 30 s exercise performance test, the patients were asked to do 1–2 fewer repetitions than performed during the maximal performance test for the rest of that week. Over each following week, the patients were encouraged to try to increase their repetitions by at least 1 or 2 per exercise.

### 3.4 Daily congratulation message and humorous exercise “meme”

After entering data for the day, a brief congratulations message appeared on the screen as well as a humorous PA-related image (i.e., meme). We chose to present congratulatory and humorous messages at the end of exercise session in keeping with the tenets of the peak-end rule, that posits the remembered quality of experiences are rated based, in part, on how they end (Do et al., 2008).

### 3.5 Weekly “social proof” email

At the end of each week, all patients received an anonymized email detailing how often the other patients had completed the exercise prescription, what the longest daily streak was, and comments that other patients provided. Similar social proof approaches have effectively motivated a range of behaviors, including reduced alcohol intake and improved hand hygiene (Dotson et al., 2015, Gaube et al., 2020). In addition, at the end of each week during the first month, patients responded to a single open-ended question regarding how they felt the exercise prescription was going and whether they had any concerns.

### 3.6 Primary care physician reports

**Fig. 1:**
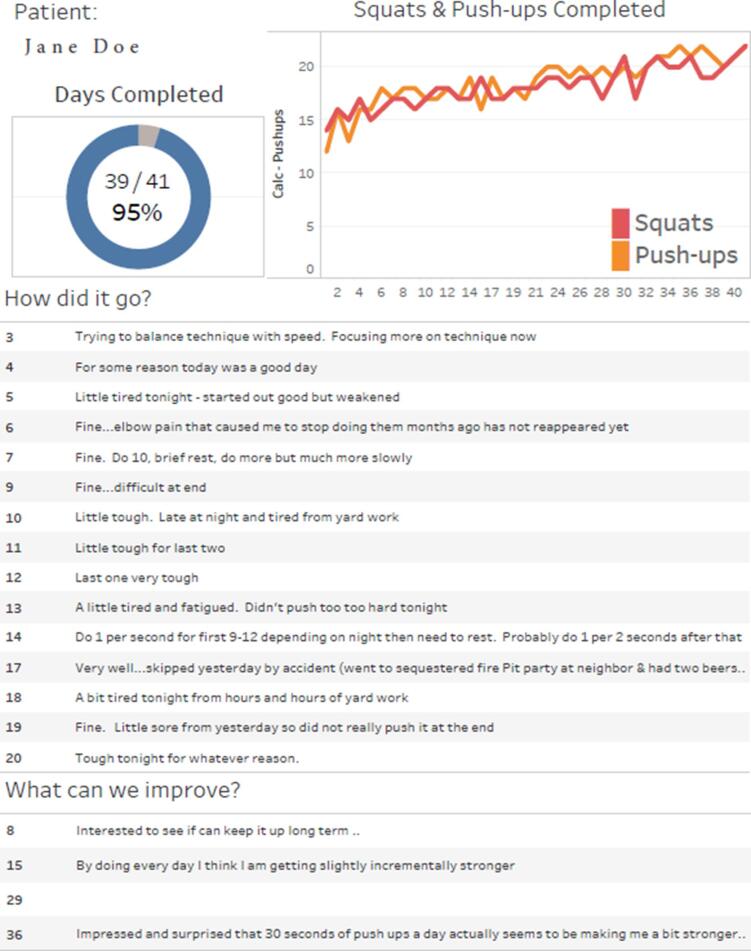
Example of a patient progress report provided to the PCP.

When a visit with a patient was scheduled, a report on the exercise adherence and performance data of the patient was securely communicated to the PCP for discussion. We used the data visualization program Tableau (Seattle, WA) to create visual reports that could be interpreted quickly by the provider (see Fig. 1 for example PCP report).

## 4 Measures

Patients responded to all measures and entered their daily push-up and squat performances using electronic surveys via a secure database (REDCap (Harris et al., 2009)).

### 4.1 Baseline physical activity

PA and sedentary behavior at baseline were determined using the moderate-to vigorous-intensity leisure-time PA and time spent sedentary (i.e., sitting) items from the International Physical Activity Questionnaire (IPAQ). Considering the inherent limitations of self-report, the IPAQ has shown acceptable concurrent validity with objectively-measured PA (*r =* 0.43-0.58; (Cleland et al., 2018)) and test–retest reliability (*r =* 0.50-0.98) among adults.

### 4.2 Perceived exertion

Each day immediately following the exercises for the first month, perception of effort was recorded using the 10-point Category-Ratio scale (CR-10; (Noble et al., 1983)). CR-10 scores less than 5 are considered moderate-intensity, with approximate changes in heart rate of 40–60 beats-per-minute (RIEBE et al., 2015). The CR-10 has shown good concurrent validity among adults with heart rate (*r =* 0.75*)*, oxygen uptake (0.77), and ventilation (0.74) (Coquart et al., 2009).

### 4.3 Affective responses to exercise

Each day immediately following the exercises for the first month, patients were asked to recollect their affective responses during exercise using the Feeling Scale (FS; (Hardy and Rejeski, 1989). The FS is a single-item, 11-point bipolar scale designed to measure feelings of pleasure and displeasure during exercise. We asked patients to rate, on a scale of −5 (Very bad) to + 5 (Very good), “How did you feel during today’s workout?”

### 4.4 Exercise self-efficacy

Exercise self-efficacy was measured using the 5-item Self-Efficacy for Physical Activity (SEPA) scale (Marcus et al., 1992). The internal consistency in the current sample was poor (*α* = 0.36) and, therefore, these values were not used in subsequent analyses.

## 5 Analysis

Maximal 30 s push-up and squat performance data were extracted from baseline and the last days of weeks-12 and-24. When week-12 and/or-24 data were missing, we assumed no change in exercise performance and imputed the missing data by carrying the last observation forward. 7 of 24 patients were missing data from the last day of week-12 and/or-24 but completed, on average, 46.7 ± 32.5 of all other daily exercise sessions. Of these 7 patients, 6 provided data only at the maximal 30 s baseline test and 1 provided data at the baseline and week-12 but not week-24 maximal 30 s tests.

To analyze changes in maximal 30 s exercise performance at baseline, week-12, and week-24, we used repeated-measures analysis of variance (ANOVA). Where violations of sphericity occurred, degrees of freedom were adjusted using the Greenhouse-Geisser method (indicated by degrees of freedom with decimals). In the case of significant effects, Bonferroni post-hoc tests were performed. Finally, we used Pearson product–moment correlations to examine the relationship between exercise adherence and exercise affective responses, ratings of perceived exertion, and baseline MVPA. Because we performed 15 tests of probability during the post-hoc tests and correlational analyses, to reduce the probability of Type I error due to multiple comparisons, family-wise *α* was Bonferroni-adjusted to 0.003.

## 6 Results

Of 57 patients 60 years of age or older sent the prescription by their PCP, 24 (42%) began completing the daily exercises (*n*_male_ = 16, *n*_female_ = 8, *mean*_age_ = 71.6yrs; see Table 1 for sample statistics). Among those who started the exercise prescription, the number of days completed (of 168 possible) was 114.2 ± 59.8. 18/24 patients (75.0%) completed at least half of the possible daily exercise sessions (84 days) and 14/24 patients (58.3%) completed at least 80% (134 days).

**Table 1:** Patient demographic, PA, and medical characteristics.

Patient Characteristic	*n*	Mean	SD	Min	Max
Age	24	71.6	8.9	61	94
BMI	23	28.6	4.8	19.9	39.8
Normal Weight	6				
Overweight	10				
Obese	8				
Weekly Moderate-Intensity PA (mins)	19	41.6	35.6	0	120
Weekly Vigorous-Intensity PA (mins)	19	32.5	54.9	0	210
Daily sitting (hours)	17	6.2	2.6	3.0	13.0

Patient Medical Conditions	Yes	No
Diabetes	4	20
High blood pressure	14	10
Cardiovascular disease	4	20
Hip replacement	3	21
Knee replacement	1	23
Rotator cuff surgery	3	21

### 6.1 Adverse events and patient acceptability

The preliminary results suggest that the daily exercise prescription was safe and well-accepted by most patients. The 2 patients who formally opted-out of the prescription by contacting the PCP reported regular exercise participation and did not wish to add more. Some of the qualitative comments by patients indicated that the push-ups were difficult, that modifications were often necessary, and that the brief nature of the exercises may have served as a motivating factor for their adherence. Several qualitative comments attesting to the acceptability of the exercise prescription are summarized in Table 2. The patients reported 3 adverse events to the PCP over the course of 24-weeks, of which 2 were deemed to be “possibly” or “likely” related to the exercise program. These included experiencing shoulder pain during push-upsand dull headaches following exercise. A third reported an unrelated previous back sprain that was aggravated by the exercise program. This patient did not report continuing symptoms after beginning to use modified push-ups and completing fewer repetitions.

**Table 2:** Examples of patient qualitative responses regarding the exercise prescription.

Response Theme	Patient examples
Exercise difficulty	“I'm amazed at how hard I'm breathing after only 1 min of exercising. So, I guess that's good.” – Male, 65 years old “They (push-ups) are very hard and not fun” – Female, 73 years old “Push-ups still difficult but a little less exhausting today” – Male, 73 years old “Push ups were the hardest of the two exercise.” Male, 78 years old
Having a routine helps	“I love having a routine and this accountability” – Male, 94 years old “I like it. Emails every day I don't forget.” – Female, 61 years old
Modifications are often needed	“My left shoulder really hurt when I tried to do regular pushups. Did modified ones instead.” – Male, 62 years old “Still modified, still difficult, but was able to do two more.” – Female, 68 years old
Patient satisfaction	“I'm happy that I'm doing this everyday” – Female, 73 years old “Keeping me active. I like it.” – Male, 62 years old “Look forward to it each day and like the challenge of pushing myself.” – Male 73 years old “I like that its quick and I don't have to go to great lengths to get some exercise in each day.” – Female, 64 years old

### 6.2 Relationships between demographic, medical, baseline physical activity and exercise adherence

The age, biological sex, BMI, medical history, and PA and sedentary behavior level at baseline of the patients were not associated with program adherence or performance.

### 6.3 Relationships between psychophysiological variables and exercise program adherence

The mean recalled perceived exertion of the exercises over the first month was 4.2 ± 1.6, corresponding to a “moderate“ to ”hard” level of intensity. Over the first month, the mean recalled session affective responses among the patients was 1.9 ± 1.5 on an 11 point scale from −5 to +5, suggesting that the patients remembered feeling “neutral” to “fairly good” during exercise. However, these variables were unrelated to exercise adherence or maximal 30 s performance.

### 6.4 Changes in maximal 30 second push-up performance

**Fig. 2:**
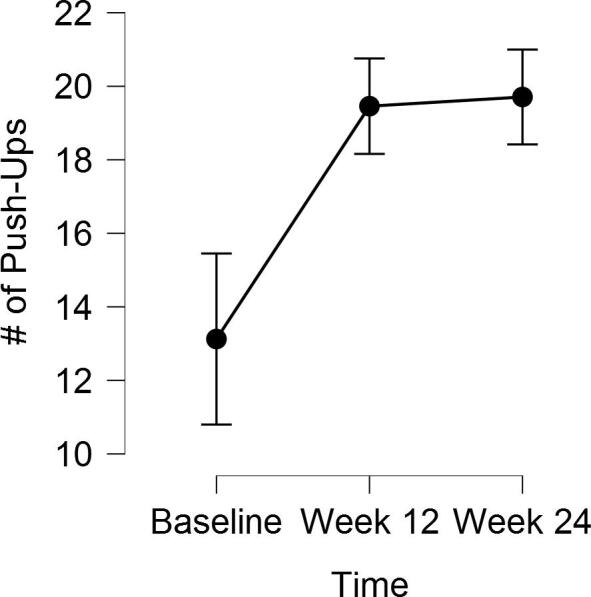
Changes in overall 30 s push-up performance over time. Error bars represent 95% confidence intervals. Note that confidence intervals were calculated via the formulae provided by Morey (Morey, 2008).

Maximal push-up performance over 30 s increased significantly over time (*F*(1.16, 26.62) = 20.40, *p* < .0001, *η* = 0.47; see Fig. 2). Bonferroni post-hoc tests revealed significant changes in overall group-level 30 s push-up performance from baseline to week-12 (*mean difference baseline-to week-12 =* 6.3, 95% CI = 2.7 – 9.9) and from baseline to week-24 (*mean difference baseline-to week-24 =* 6.6, 95% CI = 2.9 – 10.2) but not from week-12 to-24 (*p* = .58). However, those who reported using modified push-ups did not increase their maximal 30 s push-up performance over time (*p* = .008; see Table 3).

**Table 3:** Maximal 30 s push-up and squat performance and mean changes over time.

	Baseline (mean ± SD)	SMD Baseline → Week-12 (95% CI)	Week-12 (mean ± SD)	SMD Week-12 → Week-24 (95% CI)	Week-24 (mean ± SD)	SMD Baseline → Week-24 (95% CI)
Overall 30 s Push-Up Performance	13.1 (7.1)	6.3 (3.4–9.2)*	18.1 (10.2)	0.3 (−0.7–1.2)	19.7 (9.6)	6.6 (3.7–9.5)*
Unmodified 30 s Push-Up Performance	12.9 (7.6)	7.9 (3.1–12.6)*	19.2 (13.1)	0.1 (−1.5–1.3)	20.3 (12.2)	7.7 (3.1–12.3)*
Modified 30 s Push-Up Performance	12.7 (6.7)	4.2 (1.6–6.8)	16.1 (4.0)	0.8 (−1.8–3.4)	18.8 (4.3)	5.0 (−1.5–8.5)
30 s Squat Performance	17.4 (5.8)	5.9 (2.9–8.9)*	21.5 (6.4)	0.4 (−2.4–1.7)	23.0 (6.1)	5.5 (2.9–8.1)*

* = p < .003.

### 6.5 Changes in maximal 30 second squat performance

**Fig. 3:**
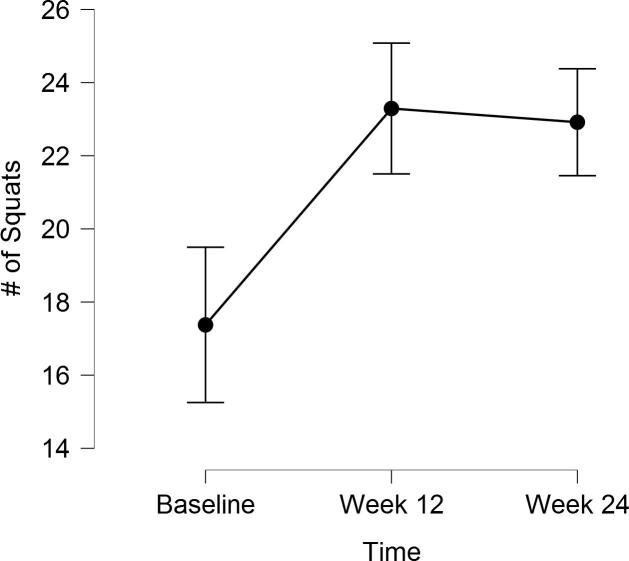
Changes in 30 s squat performance over time. Error bars represent 95% confidence intervals. Note that confidence intervals were calculated via the formulae provided by Morey (Morey, 2008).

Maximal squat performance over 30 s increased significantly over time, *F*(2, 46) = 14.31, *p* < .0001, *η*  *=* 0.38 (see Fig. 3). Bonferroni post-hoc tests revealed significant changes in 30 s squat performance from baseline to week-12 (*mean difference baseline-to week-12 =* 5.9, 95% CI = 2.2 – 9.6) and from baseline to week-24 (*mean difference baseline-to week-24 =* 5.5, 95% CI = 2.3 – 8.8) but not from week-12 to-24 (*p =* .71, see Table 3).

## 7 Discussion

The purpose of this quality improvement initiative was to evaluate the feasibility and preliminary impact of a daily brief functional exercise program prescribed to older adults by their PCP. Our data suggest that both adoption (i.e., 42% of patients started the exercise prescription) and adherence were high. The mean number of days completed of 168 days was approximately 114 and 75% completed at least one-half of possible exercise sessions. 71% of patients continued to report completing the exercises on at least some days after 24-weeks, despite including no additional personal coaching or support. These proportions are several times larger than the percentage of adults who adopt free exercise such as Silver Sneakers and Enhance Fitness (e.g., only 3.8% of 55,127 older adults attended an exercise session (Greenwood-Hickman et al., 2015)). In addition, we observed significant overall group increases for maximal push-ups (~6.5) and squats (~5.7) in 30 s over time. At the same time, however, those patients (*n* = 10) who reported using push-up modifications did not improve their push-up performance significantly over time. This result is understandable, given the relatively lower amount of body mass necessary to lift when doing push-ups from the knees or on a staircase and our small sample size. Practitioners who implement similar programs among their patients should be prepared to observe a slower rate of improvement among patients who use modifications. Encouraging patients to progress to more difficult modifications may increase the rate at which changes in performance are observed. Finally, the overall group performance for both push-ups and squats in 30 s plateaued after week-12. This plateau was likely related to only 30 s being allotted to complete each exercise. That is, once a certain number of repetitions were reached, patients could complete no additional repetitions within the 30 s time limit while still maintaining proper form.

While it is not entirely clear why adoption and adherence to the current program was high, framing the program as a prescription coming directly from the PCP of the patients that would be discussed during visits may have led to pressure to adhere. Indeed, “simply notifying patients that follow-up will occur seems to be a powerful motivating factor” to adhere to PCP directives (Whitlock et al., 2002). Still, the COVID-19 pandemic should also be considered. The first emails were sent to patients from their PCP in April 2020 while the county in which the PCP is located was under stay-at-home orders. These orders were lifted in June 2020. It is possible patients were more interested in the exercise prescription because they had little else to do while mandated to stay-at-home. Indeed, survey research firms have reported that phone survey completion rates have increased during the COVID-19 pandemic (Russonello and Lyall, 2020).

## 8 Limitations

Although this program has a number of strengths, including the element of a direct PCP “prescription”, its ease of administration, as well as its setting in primary care, there are also several limitations. First, the work was not formal research, meaning objective measures of physical performance and function were not completed, and measurement errors, such as in repetition counting inaccuracy or improper exercise form might have occurred. Second, adherence was measured via self-report, which is notoriously inaccurate for estimating PA (Carlson et al., 2010, Tucker et al., 2011). However, in this program the accuracy of self-report may have been improved as the patients reported their exercise performance on the same instrument that demonstrated their completion of the prescription to their PCP. Moreover, reducing the period of recall to less than 24 h has been shown to improve the accuracy of self-report (Matthews et al., 2019). The current exercises were also only prescribed to one group of patients under the care of the same PCP, whose research has focused on PA, and who completed the voiceovers and videos. The results may not generalize to other providers with less explicit interest in PA promotion. In addition, at baseline, the MVPA levels of some patients were above the national average, though we did not measure the frequency at which the patients participated in strength training. Future work will be necessary to determine the impact of this program among patients who are less active. Finally, we cannot infer whether the program was effective based only on a single-group of patients, and subsequent formal controlled research studies should be performed to further examine efficacy.

## 9 Conclusion

The results from this quality improvement initiative suggest that one-minute of daily functional exercise prescribed to older adult patients by their PCP was well-accepted with, few adverse events clearly related to the exercises program, had a good rate of adoption and 24-week adherence, and led to significant changes in 30 s push-up and squat performance. Primary care initiatives such as this one could help to identify the shortest possible exercise program that can be prescribed by a physician that leads to the desired clinical effect among his or her patients (i.e., good adoption, adherence, and a reduction in future losses of physical function and/or disability). This is identical to the way other approaches are devised in medicine, where the dose and delivery mechanism are chosen to optimize adherence and beneficial effects, while minimizing adverse effects. Considering the costs of travel and fitness center memberships and low frequencies of use even when provided freely, this home-based exercise prescription may present an attractive alternative for older adults. In addition, especially for older adults, the ongoing COVID-19 pandemic has made visits to exercise centers risky. This exercise prescription can be easily delivered electronically and may allow older people to maintain or reduce fitness losses during periods when normal exercise facilities are inaccessible or when home exercise is preferred. Given these results, formal controlled research should be carried out to understand whether similar programs can be successfully disseminated among the patients of other physicians.

## Declaration of Competing Interest

Christopher Sciamanna has an investment, such as stock, in a company which has begun to investigate the possibility of creating a business that provides exercise programs. All other authors declare that they have no known competing financial interests or personal relationships that could have appeared to influence the work reported in this paper.
