# Supplementary material for: Feasibility and impact of a 1-minute daily functional exercise regimen prescribed to older adults by their primary care physician
Source: Prev Med Rep. 2021 Jan 4;21:101307. doi: 10.1016/j.pmedr.2020.101307 (PMC7820134; doi:10.1016/j.pmedr.2020.101307)
Supplement: Supplementary data 1 [file mmc1.pdf]

Tutorial in Quantitative Methods for Psychology
2008, Vol. 4(2), p. 61-64.

Confidence Intervals from Normalized Data: A correction to Cousineau (2005)

Richard D. Morey
University of Missouri-Columbia

Presenting confidence intervals around means is a common method of expressing uncertainty in data. Loftus and Masson (1994) describe confidence intervals for means in within-subjects designs. These confidence intervals are based on the ANOVA mean squared error. Cousineau (2005) presents an alternative to the Loftus and Masson method, but his method produces confidence intervals that are smaller than those of Loftus and Masson. I show why this is the case and offer a simple correction that makes the expected size of Cousineau confidence intervals the same as that of Loftus and Masson confidence intervals.

Confidence intervals (CIs) are a staple in the presentation of psychological data because they allow researchers to quickly gage the amount of uncertainty in data (Rouder & Morey, 2005). For within-subjects designs, there are several approaches to creating confidence intervals. For a given design it may not be clear which to choose. Consider a simple within-subjects design with two conditions, a pre-test and post-test. For this design, there are multiple methods of generating confidence intervals. I will discuss each in turn.

Approaches to confidence intervals

The standard way to build confidence intervals is to compute the standard error of the mean for each condition, and multiply it by the appropriate t-distribution quantile. In order to make this concrete, Table 1 lists hypothetical data for N = 10 participants. A paired t-test reveals a significant effect of condition $(\mathrm{MSE} = 5.19, t(9) = 3.65, p = .005,$ partial-$\eta^2 = .60)$. Means are shown as points in Figure 1. To build a 95% confidence around each mean, one computes the standard error $(SD/\sqrt{10})$ and multiplies this by $t_{.975}(9) = 2.26$. This gives the size of the CI around the mean. The resulting confidence intervals are labeled "A" in Figure 1. The size of the confidence intervals is larger than what might be expected from the results of the t-test. The t-test indicates a large, significant effect, but the confidence intervals overlap. The reason for this is that each observation reflects three sources of variance: the fixed effect of condition, the random ability of each participant, and random error. The size of the CIs labeled "A" are determined by both participant variance and error variance. The paired t-test, in contrast, accounts for participant variability in computing the significance of the effect.

Loftus and Masson (1994) suggested that confidence intervals in within-subject designs be based on the denominator Mean Square (MS) from the appropriate ANOVA analysis. Because the MS is an unbiased estimate of the appropriate error variance (Moser, 1996; Loftus & Masson, 1994), using the MS would yield a confidence interval uncontaminated by variance from participants. To build Loftus and Masson CIs, one computes a within-subjects standard error by dividing the ANOVA denominator MS by N and taking the square-root. Using this standard error in place of the standard error of each condition, then multiplying by the appropriate t quantile, yields a within-subjects confidence interval. The CIs labeled "B" in Figure 1 are Loftus and Masson confidence intervals.

Address correspondence to Richard D. Morey, Department of Psychological Sciences, 210 McAlester Hall, University of Missouri, Columbia, MO 65211 or to moreyr@missouri.edu.
This research is supported by NSF grant SES-0351523 and NIMH grant R01-MH071418.

Table 1. Sample results used in Figure 1.

Participant #	Pretest	Posttest
1	59.4	64.5
2	46.4	52.4
3	46.0	49.7
4	49.0	48.7
5	32.5	37.4
6	45.2	49.5
7	60.3	59.9
8	54.3	54.1
9	45.4	49.6
10	38.9	48.5
Mean	47.74	53.43
SD	8.60	7.25

These confidence intervals are much tighter and better reflect our conclusions from the t-test analysis.

Loftus and Masson's method has two primary drawbacks: First, all CIs are the same size, so any heterogeneity of variance is hidden; second, it may be inconvenient to extract the appropriate MS and force a statistical program, such as SPSS, to use it to build CIs. Cousineau (2005) suggested another method for constructing confidence intervals in within-subjects designs. Cousineau's method is simple and seems reasonable: *normalize* the data by subtracting the appropriate participant's mean performance from each observation, and then add the grand mean score to every observation. Then, use the normalized data to build confidence intervals using the standard method described above.

In notation, the Cousineau method can be described as follows. Let y_{ij} be the ith participant's score in the jth condition ($i = 1, \ldots, N;\ \ j = 1, \ldots, M$). Then define the normalized observations z_{ij} as

$$z_{ij} \quad = \quad y_{ij} - \sum_{j=1}^{M} y_{ij} + \sum_{i=1}^{N}\sum_{j=1}^{M} y_{ij}. \qquad (1)$$

The variances of the resulting normalized values in a condition, and thus the size of the CIs, no longer depend on the participant effects (Loftus & Masson, 1994; Masson & Loftus, 2003). The intervals labeled "C" in Figure 1 were generated by Cousineau's method. The primary benefit of the normalization method over basing the CIs on the MS is ease of use and intuitiveness, and Cousineau provides SPSS code to easily generate the normalized scores.

Inconsistency between approaches

Examination of Figure 1 reveals a mismatch between the size of Loftus and Masson style CIs and CIs based on normalized data. If the size of both CIs is based only on the error variance, why are normalized CIs smaller than those based on the MS? The MS from the ANOVA analysis has properties that are well-understood in linear model theory. There is very little discussion in the literature, however, on the properties of CIs built from normalized scores. If CIs based on normalized data are to be useful, it is important to understand the reason for the mismatch between Loftus and Masson CIs and Cousineau CIs.

The reason that CIs based on normalized scores will be smaller on average is that normalizing scores induces positive covariance between scores within a condition. Because all the data is used in the computation of a single normalized observation (see Eq. 1, the observations become correlated. If scores are correlated, the sample variance will be biased low, because positively correlated scores will not vary as much as expected from one another. Thus, even though both Loftus and Masson CIs and Cousineau CIs are functions of error variance alone, Loftus and Masson CIs are based on an unbiased estimate, while Cousineau CIs are based on an estimate biased to be small. All CIs generated from normalized scores will be "too small" with respect to Loftus and Masson CIs. Loftus and Masson (1994) noted this in their Appendix, and a general proof of this fact is provided in the Appendix of this manuscript.

The size of Cousineau CIs relative to Loftus and Masson CIs is a function of the number of within-subjects conditions. The expected value of the sample variance in a condition of normalized data is

$$E\left[\frac{SS_j}{N-1)}\right] \quad = \quad \sigma_\epsilon^2 \frac{M-1}{M}, \qquad (2)$$

where σ_ϵ^2 is the error variance, $SS_j$is the sum of squares for condition$j$, and M is the number of within-subjects conditions. The worst case bias occurs for $M = 2$, and the bias decreases as M increases.

An easy correction

Eq. 2 suggests a way of correcting the bias of Cousineau CIs. Compute the sample variance of the normalized data as Cousineau suggests, then simply multiply the sample variances in each condition by $M/(M-1)$. Multiplying by this correction factor will bring the size of Cousineau CIs into line with Loftus and Masson CIs. For $M = 2$, the resulting corrected CIs will be exactly the same size as the Loftus and Masson intervals. The corrected Cousineau CIs for the hypothetical data are represented by "B" in Figure 1. For $M > 2$, corrected CIs will not be exactly the same size in general, but their expected size is the same as Loftus and Masson CIs.

This simple correction also holds for more complicated designs. It can accommodate any number of fixed effects; simply replace M with $\prod_{p=1}^{P} M_p$, where P is the number of fixed factors, and M_p is the number of levels of the pth fixed

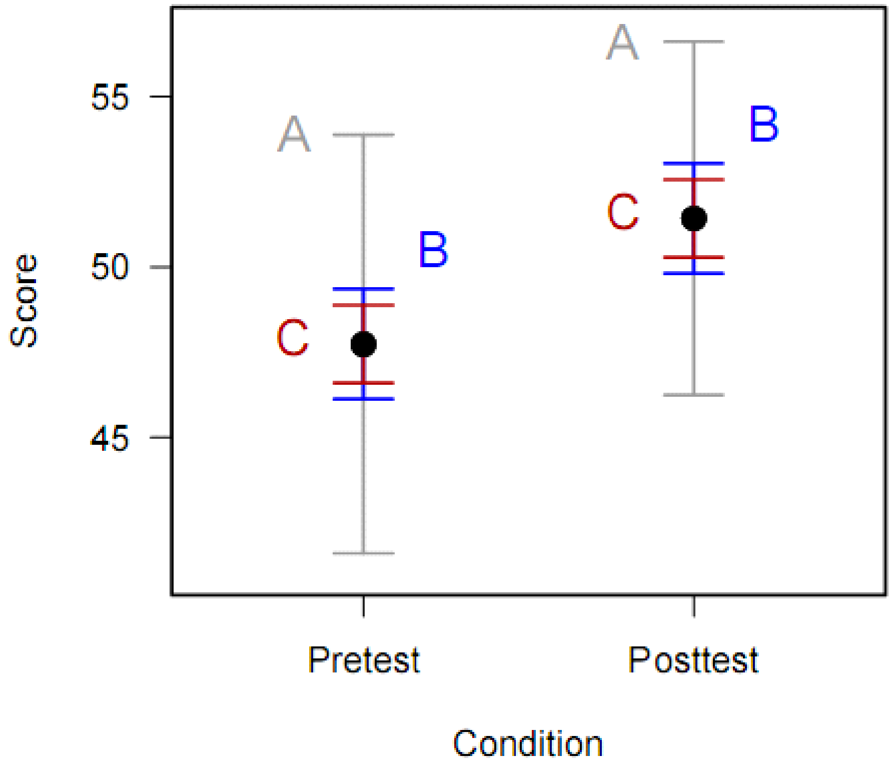

Figure 1. *Means from hypothetical data and several possible 95% CIs on the means. A: CIs from standard errors of each condition considered separately. B: CIs from ANOVA MSE. C: Cousineau (2005) CIs from normalized data.*

factor. If there are between-subject factors, each level of the between-subject factor should be considered separately. Using this simple correction, the ease-of-use of the normalizing approach is retained, while also retaining the expected size of the Loftus and Masson intervals.

General Discussion

The use of within-subjects confidence intervals in data presentation is an important way of communicating the amount of uncertainty in data. In within-subjects designs, a researcher may desire CIs whose size accords more with the result of inferential tests. Loftus and Masson (1994) and Cousineau (2005) provide methods of building these confidence intervals. Cousineau's method has two advantages: first, it is easy to compute; second, it may show violations of homogeneity of variance (Cousineau, 2005).

Understanding the properties of the methods used to create CIs is a vital part of understanding graphical presentations of data. I have shown that within-subjects confidence intervals based on normalized data are small relative to Loftus and Masson within-subjects confidence intervals. Luckily, however, the bias in normalized CIs is easily corrected; the simple correction suggested above will make the expected size of Cousineau CIs the same as the commonly-used Loftus and Masson CIs.

References

Cousineau,D. (2005).Confidence intervals in within-subject designs:Asimpler solution to Loftus and Masson's method. *Tutorials in Quantitative Methods for Psychology*, *1*, 42-45.

Loftus, G.R., & Masson, M.E.J. (1994). Using confidence intervals in within-subject designs. *Psychonomic Bulletin and Review*, 1, 476-490.

Masson, M.E.J., & Loftus, G.R. (2003). Using confidence intervals for graphically based data interpretation. *Canadian Journal of Experimental Psychology*, 57,203-220.

Moser,B.K. (1996). *Linear models: A mean model approach*. San Diego, CA: Academic Press.

Rouder,J., & Morey, R.D. (2005). Relational and arelational confidence intervals:A comment on Fidler et al. (2004). *Psychological Science*, 16, 77-79.

Appendix

In this appendix I prove the main result that CIs based on normalized data are based on biased estimates of error variance. Consider the model

$$\begin{aligned} y_{ij} &= \mu + \alpha_i + \beta_j + \epsilon_{ij}, \\ \alpha_i &\overset{indep.}{\sim} \text{Normal}(0, \sigma_\alpha^2), \\ \epsilon_{ij} &\overset{indep.}{\sim} \text{Normal}(0, \sigma_\epsilon^2), \end{aligned}$$

where the $\alpha_i (i = 1, \ldots, N)$ are random participant effects and $\beta_j (j = 1, \ldots, M)$ are fixed condition effects (ie,$\sum_j \beta_j = 0$), and all ϵ_{ij} and α_i are mutually independent.

Let the multivariate normal data vector $\mathbf{y}$ be

$$\begin{bmatrix} y_{1,1} \\ y_{1,2} \\ . \\ . \\ y_{1,M} \\ y_{2,1} \\ . \\ . \\ y_{N,M} \end{bmatrix} \tag{3}$$

It is obvious that the covariance matrix for $\mathbf{y}$ given the model is

$$\mathbf{\Sigma_y} = \mathbf{I}_N \otimes [\sigma_\alpha^2 \mathbf{J}_M + \sigma_\epsilon^2 \mathbf{I}_M], \tag{4}$$

where $\mathbf{I}_N$ is the $N \times N$ identity matrix, $\mathbf{J}_N$is an $N \times N$ matrix of all 1, and $\otimes$ is the Kronecker product operator.

Normalization of the vector $\mathbf{y}$ (Cousineau, 2005) is accomplished by multiplying the vector by

$$\mathbf{C} = \left(\mathbf{I}_N \otimes \mathbf{I}_M \right) - \left(\mathbf{I}_N \otimes \frac{1}{M} \mathbf{J}_M \right) + \left(\frac{1}{N} \mathbf{J}_N \otimes \frac{1}{M} \mathbf{J}_M \right). \tag{5}$$

The covariance matrix of the normalized data vector $\mathbf{z} = \mathbf{Cy}$ is

$$\begin{aligned} \mathbf{\Sigma}_z &= \mathbf{C}^T \mathbf{\Sigma}_y \mathbf{C} \\ &= \mathbf{C} \mathbf{\Sigma}_y \mathbf{C}. \end{aligned} \tag{6}$$

Equation 6 follows from the fact that $\mathbf{C}$ is symmetric.

Multiplying the matrices in equation 6 yields

$$\begin{aligned} \mathbf{\Sigma}_z = & \left[\mathbf{I}_N \otimes \left(\sigma_\alpha^2 \mathbf{J}_M + \sigma_\epsilon^2 \mathbf{J}_M \right) \right] \\ & - \left[\mathbf{I}_N \otimes \left(\sigma_\alpha^2 + \frac{\sigma_\epsilon^2}{M} \right) \mathbf{J}_M \right] \\ & + \left[\frac{1}{N} \mathbf{J}_N \otimes \left(\sigma_\alpha^2 + \frac{\sigma_\epsilon^2}{M} \right) \mathbf{J}_M \right] \end{aligned} \tag{7}$$

Because we are only interested in the observations within a level of β, we only need to consider vector of length N, $\mathbf{w}$, containing the observations of interest (all those from level j of β). We can construct the $N \times N$ covariance matrix $\mathbf{\Sigma}_w$ by examination of the elements of $\mathbf{\Sigma}_z$. Let a be the on-diagonal elements of $\mathbf{\Sigma}_z$, and let b be the off-diagonal elements corresponding to the observations within a condition. Examination of equation 7 yields

$$\begin{aligned} a &= \sigma_\epsilon^2 \left(\frac{MN - N + 1}{NM} \right) + \frac{\sigma_\alpha^2}{N} \\ b &= \frac{\sigma_\alpha^2}{N} + \frac{\sigma_\epsilon^2}{MN} \end{aligned} \tag{8}$$

With these values, the covariance matrix $\mathbf{\Sigma_w}$ is

$$\mathbf{\Sigma}_w = (a - b)\mathbf{I}_N + b\mathbf{J}_N \tag{9}$$

It is also easy to show that $\mathrm{E}[w] = (\mu + \beta_j)\mathbf{1}$, where $\mathbf{1}$ is a column vector of all 1. Let $\mathbf{K}$ be the $N \times N$ centering matrix, ie

$$\mathbf{K} = \mathbf{I}_N - \frac{1}{N} \mathbf{J}_N \tag{10}$$

The sum of squares of the vector $\mathbf{w}$ is then $\mathbf{w}^T \mathbf{K} \mathbf{w}$. Because for any random vector $\mathbf{x}$ and matrix $\mathbf{A}$, $\mathrm{E}[\mathbf{x}^T \mathbf{A} \mathbf{x}] = \mathrm{trace}(\mathbf{A}\mathbf{\Sigma}) + \boldsymbol{\mu}^T \mathbf{A} \boldsymbol{\mu}$,

$$\begin{aligned} \mathrm{E}[\mathbf{w}^T \mathbf{K} \mathbf{w}] &= \mathrm{trace}(\mathbf{K}\mathbf{\Sigma_w}) + (\mu + \beta_j)^2 \mathbf{1}^T \mathbf{K} \mathbf{1} \\ &= \mathrm{trace}\left(\left[\mathbf{I}_N - \frac{1}{N} \mathbf{J}_N \right] [(a - b)\mathbf{I}_N + b\mathbf{J}_N] \right) + 0 \\ &= (a - b)(N - 1) \\ &= (N - 1) \frac{M - 1}{M} \sigma_\epsilon^2 \end{aligned}$$

Dividing by $N - 1$ to obtain the expected value of the sample variance yields $\frac{M-1}{M} \sigma_\epsilon^2$.

Manuscript received 11 November 2007
Manuscript accepted 8 May 2008
