# Supplementary material for: Feasibility and impact of a 1-minute daily functional exercise regimen prescribed to older adults by their primary care physician
Source: Prev Med Rep. 2021 Jan 4;21:101307. doi: 10.1016/j.pmedr.2020.101307 (PMC7820134; doi:10.1016/j.pmedr.2020.101307)
Supplement: Supplementary data 2 [file mmc2.docx]

**Supplementary Materials**

**Text of initial letter/email from PCP providing exercise prescription to patient.**

Hello,

Good news! New studies show that very short workouts have huge effects on strength and fitness.

So, starting today, I'm going to expect EVERY ONE of my patients to spend 1 minute exercising - after brushing teeth - each day. You may already have seen the letter that I sent to ALL of my patients.

I need you to do 30 seconds of push-ups, rest for 10 seconds and then do 30 seconds of squats. While more exercise is better, push-ups and squats build fitness and strength and reduce risk of heart attacks. You care for your mouth in 1 minute each day and now you can do great things for the rest of your body in 1 extra minute. Most people are surprised how quickly they are able to double the number of push-ups they are able to do.

To get started, please watch the short video in the link below. Also, I have set you up in an automated system to help us keep track of your progress. You'll be asked 3 short questions each day that will take 30 seconds. I'll send you a report based on your progress. If you want to opt out and not get this information, please let me know.

[Daily one minute workout](https://urldefense.com/v3/__https:/redcap.ctsi.psu.edu/surveys/?s=fcvXboVoGT__;!!Ls64Rlj6!gvybarcsYgewEq88MKkAiPk5J0rCEK8TpdQtPrzMmWvqOnukRHeWgQxXhy39R50QSYT03Ic$)

If the link above does not work, try copying the link below into your web browser:

survey url

When I see you next, I'm going to ask you "How many push-ups can you do in 30 seconds?" and I'll have the information you entered into our system. If you have any questions or think you cannot (or should not) do these exercises, please let me know.

Let's all get stronger and in better shape this year the easy way. Thanks!

Chris

**Follow-up email sent until patient response received.**

Hey, I'm just checking in. I didn't see that you tried the 1 minute workout, so I wanted to see what's going on. I know it's not perfect for all of my patients, though several dozen have started, so I wanted to see what's getting in the way of others starting.

I'm trying to make this useful, so please let me know what's getting in the way. Feel free to say, "I hate push-ups". I can handle the truth :)

Chris

**Example of daily email received by patient.**

You are now on day 58 of the One Minute Workout!

Click here to time your exercise and enter the number of push-ups and squats that you were able to do:

[Daily one minute workout](https://urldefense.com/v3/__https:/redcap.ctsi.psu.edu/surveys/?s=fcvXboVoGT__;!!Ls64Rlj6!gvybarcsYgewEq88MKkAiPk5J0rCEK8TpdQtPrzMmWvqOnukRHeWgQxXhy39R50QSYT03Ic$)

If the link above does not work, try copying the link below into your web browser:

survey url

This link is unique to you and should not be forwarded to others.

If you have any questions please me or, if urgent, can me on my cell phone at 610-585-9765.

Thank you in advance for doing your absolute best!

Chris

**Baseline, week-6 and-12 maximal test audio recording (italics indicate that this line was only used for baseline audio recording)**

Hello, today we're going to see how much you can do as in the video. The goal here is to see how many push-ups and squats you can do. I'm going to ask you to do the push-ups in the normal way, not on your knees or any other way. *And if you can do zero, that's fine. We just need to know where we're starting from.* So just give it your all and see what you can do. And remember, it's a bit of a race, so try to go faster than you would normally. With that said, let's get going.

Start with push-ups, get down and get your hands in the push-up position with your arms shoulder-width apart. All right, ready? Set. Go. 29, 28, 27, do your best to keep your body straight as an arrow, but go fast, please 22, 21, 20. Don't stop breathing. Breathe in, breathe out. Keep moving fast. This is a test, 12, 11, 10. Now let's give it your all for the final 10 seconds to see how many you can do when you give it your all 3, 2, 1, 0, alright. Congratulations. Okay. You're half done. And that was definitely the harder part of the two.

But let's move on to squats. Take a few seconds, take a few deep breaths. Now, you're going to get your feet about as wide as your shoulders, just like your hands were in push-ups and you'll mainly just be sitting down, but you're going to go up and down pretty fast. This is a race. We're really trying to see how many you can do when you give it your all. All right. So, your feet are set. Ready? Set. Go. 29, 28, 27. Remember, it's a race. You try to go fast, 23, 22, 21, 20. If your heels are coming off the ground, you're probably not sitting down far enough. 14, 13, 12, 11, 10. Alright, keep breathing, push through as many as you can do. Keep moving. 4, 3, 2, 1, 0, finished.

Right. All right. Nice work. So don't forget to give yourself credit today by entering the information on online with the email link, so I can have it when I see you and see how much better you've done. So yeah, have a great day. Nice work.

**Day after each maximal test (baseline, 12, and 24-month) audio recording**

All right. Good morning. As in the video, today we're going to start with the goal of doing one or two fewer push-ups or squats than you were able to do during the all-out test that you recently did. I don't want it to be too uncomfortable each day, or you might not want to do it at all. So if you could do 8 push-ups during the maximal test, try to get 6 or 7 done today. The first goal is to build a new habit then to get stronger and faster.

So with that said, let's get going, starting with push-ups. Remember that it's okay to do push-ups on your knees, but once you can do 15 well, we'll be helping you try to move to do them the normal way. It's also easier to do push-ups by standing at the bottom of a set of stairs and placing your hands on, say the fourth step.

And once you can do 15, move down to the third step, et cetera. It's all right. Um, get your hands in up position with your arms shoulder width apart. Ready, set, go. 29, 28, 27. All right. You're best to keep your elbows close to your body. We'll focus cause on that. 21, 20, 19, 18. All right, let's give it your all or close to it. So keep pushing and make sure you keep breathing. 11, 10, 9. 6, 5, 4, 3, 2, 1, 0. Alright. Good work. Good work. Yeah. Push-ups are the hardest part by far. But let's move on to, uh, the squats. And, take a couple seconds. Take a couple, deep breaths. So, we're going to move on to squats.

Now you're going to get your feet about as wide as your shoulders and just focus on sitting down. So your heels don't come off the ground. All right. Ready? Set. Go. 29, 28, 27. All right. If your heels are coming off the ground, you're probably not sitting down far enough. 21, 20, 19. And remember, it's a race you're trying to go fast all the way down all the way up, all the way down all the way up. 13, 12, 11, 10. All right. Keep breathing. You are almost done. Don't forget to breathe. 5, 4, 3, 2, 1, Finished, alright! Good job. Nice work. Don't forget to give yourself credit by entering the information on the link you got so I can have it when I see you and we can see how much you've progressed over time.

So, all right. Have a good rest of the day. Nice work.

**Daily countdown audio recording**

All right. Good morning. I hope you're staying safe in COVID isolation. I'm recording this in my basement and other than a 10-minute grocery visit and all the driving to the med center this week to sign papers, I've been living in a nice prison. So hey, let's let get our little workout done. Starting with push-ups. Get your hands in push up position, arms shoulder width apart. Ready, set. Go. 29, 28, 27. All right, today, let's do your best to keep your back straight. 21, 20, 19, 18. And don't forget to keep breathing. Breathe. Breathe. 13, 12, 11, 10. Remember the last 10 seconds is the part that really counts. So keep your efforts strong 5, 4, 3, 2, 1, done. Right? Good job. Good job. All right. Take a, take a couple of seconds to catch your breath. Uh, not too long. We'll keep the heart rate up nice, which is good. It's only one minute at a day, so let's make it count.

Let's switch over to squats. So, get in squat position, get your feet shoulder width apart. And, and everybody ready? Set. Go. 29, 28, 27. All right. Remember, it's a race. You're trying to go fast up, down, up, down about as fast as you can. 20, 19, 18, 17. And if your heels are coming off the ground, you're probably not sitting down low enough, 10, 9, 8. Let's keep going, keep breathing. And you are almost done. 4, 3, 2, 1. Done. Alright. Good job. Great work today.

Hey, all right. The good news of the day is that I am pretty sure our COVID cases are declining. I volunteered to start contact tracing program and I supervise several dozen medical students doing this each day. Every day, I get the master list of people diagnosed with COVID at the med center and for what I can see, there's a huge number around the first week in April that was steadily building and then it's really been going down ever since. So I hate to predict the future too much, but it is similar to the rest of the US trends where the number of cases is no longer rising. So with that, uh, let's hope that trend continues have a great day and stay safe.
